# Ubiquitin specific protease 7 maintains pluripotency of mouse embryonic stem cells through stabilization of β-catenin

**DOI:** 10.3906/biy-2108-45

**Published:** 2021-11-17

**Authors:** Taha Bartu HAYAL

**Affiliations:** Yeditepe University, Department of Genetics and Bioengineering, Faculty of Engineering, İstanbul, Turkey

**Keywords:** Ubiquitination, pluripotency, USP7, P5091, embryonic stem cells, Wnt/β-catenin

## Abstract

Embryonic stem cells (ESCs), which are derived from the undifferentiated inner cell mass of the embryo, can differentiate every cell type of the body regarding their pluripotency. Therefore, human or mouse ESCs can be used as an unlimited cell source for numerous researches or therapeutical approaches. However, pluripotency maintenance of ESCs during in vitro culture is challenging because of their endless differentiation capacity. In the current study, the effect of USP7 on pluripotency maintenance of mouse ESCs (mESCs) has been investigated with the help of cell viability assay, morphological analysis, alkaline phosphatase (ALP) staining, qPCR analysis, and Western Blotting. 600 nM P5091 application, which showed no significant toxicity in mESCs, increased the total ubiquitinated protein amount as a proof of the accomplishment of proper USP7 inhibition. Morphological analysis and ALP activity evaluation indicated that dual inhibition of GSK3 and MEK together with leukemia inhibitory factor (LIF) treatment protects the pluripotency in presence of active USP7 enzyme. Yet, inactivation of USP7 reduced the ALP activity and altered the cell morphology in each treatment group. This morphological change and decreased ALP activity refer to differentiated mESCs. These findings were supported by gene expression and protein analysis. Gene expressions and protein amounts of pluripotency related Oct4, Nanog, c-Myc, Sox2 and Klf4 transcription factors decreased significantly after USP7 inhibition. Together with this observation, a remarkable reduction in β-Catenin expression was also noticed. It was also observed that USP7 inactivation shortens the half-live of β-Catenin and GSK3β proteins. This study demonstrates that USP7 activation is crucial for proper pluripotency maintenance, which is provided through β-Catenin stabilization.

## 1. Introduction

The number of researches that focused on embryonic stem cells (ESCs) is increasing because of the endless differentiation potential of ESCs. These researches provide a variety of possibilities for developing better techniques to differentiate ESCs into any cell type. For instance, from the perspective of regenerative medicine, ESCs can be used as an unlimited source for various tissue replacements (Zhu et al., 2019). Keeping ESCs at pluripotent state is crucial but challenging due to their high differentiation capacity. The regulation of pluripotency maintenance of ESCs is controlled by several transcription factors such as Oct4, Sox2, and Nanog (Do and Schöler 2009). Furthermore, cDNA microarray studies showed that those transcription factors are important for regulation of different pathways, such as Wnt/β-catenin pathway (Cheng et al., 2015).

The Wnt gene family is responsible for several glycoprotein secretions which can be used as ligands to promote numerous signaling pathways. Thus, Wnt signaling plays a crucial role in adults as well as it does in embryonic development (Clevers, 2006). There are three types of Wnt pathways: canonical Wnt pathway, noncanonical planar cell polarity pathway, and noncanonical Wnt/Ca^+2^ pathway. All of the pathways are triggered by the attachment of Wnt ligand to the corresponding receptor (Frizzled family receptor) on the cell membrane (Adamska et al., 2010). The planar cell polarity pathway of Wnt signaling is crucial for cytoskeleton organization through the activation of various proteins (Sebbagh and Borg 2014). In addition to that, the noncanonical Wnt/Ca^+2^ pathway plays a critical role in actin polymerization (Komiya and Habas 2008). Wnt/Ca^+2^ pathway also has a huge impact on cellular adhesion and migration capability of cells (Kumar et al., 2015). The downstream of the most studied Wnt gene family member, Wnt/β-Catenin pathway, was previously correlated with the pluripotency maintenance of mouse and human ESCs (ten Berge et al., 2011). In this pathway, activated Wnt receptor triggers Dvl protein, which is responsible for the inhibition of destruction complex formation (Kimelman and Xu 2006). Axin and GSK3β proteins as crucial members of destruction complex, plays a critical role in proteasomal degradation of β-Catenin protein (Wu and Pan 2010). Therefore, Wnt pathway aims to rescue β-Catenin from ubiquitination and proteasomal degradation (Gwak et al., 2006; Chen et al., 2020). It is known in the literature that Wnt signaling is also required for proper reprograming of somatic cells (Lluis et al., 2008). Furthermore, several studies on Wnt signaling demonstrated that it is critical not only for pluripotency maintenance but also in differentiation process of stem cells. Thus, the real impact of Wnt/β-Catenin pathway is its effect on cellular homeostasis of stem cells (Davidson et al., 2012). Therefore, the regulation of β-Catenin protein, which is controlled by ubiquitin-proteasome system (UPS), is highly important for cellular homeostasis (Gao et al., 2014).

Ubiquitination, which can be defined as tagging unwanted proteins for proteasomal degradation, was previously correlated with pluripotency maintenance of ESCs (Suresh et al., 2016). Ubiquitination process of proteins can be reversed for rescuing proteins from proteasomal degradation. This process is called deubiquitination and several deubiquitinases (DUBs) were identified as a key player in the stabilization of proteins (Mevissen and Komander 2017). Ubiquitin specific protease (USP) deubiquitination family member USP7 controls several cellular mechanisms through the stabilization of numerous proteins (Nicholson and Suresh Kumar 2011). Previously, USP7 was correlated with neural progenitor cell maintenance due to rescuing repressor element 1-silencing transcription factor (REST) from proteasomal degradation (Huang et al., 2011). In addition to this, recent studies have shown that USP7 deubiquitination enzyme is also important in pluripotency maintenance of human ESCs with its ability to stabilize Nanog, Sox2 and Oct4 (Wei et al., 2019).

Identification of ubiquitination mechanisms that is important in ESCs maintenance might be very useful for in vitro studies as well as for future cellular therapy approaches. Therefore, the current study aims to identify a novel target for USP7 enzyme in pluripotency maintenance of mouse ESCs. The effect of USP7 on mouse ESCs was evaluated by inactivation of USP7 enzyme activity using small molecule inhibitor, P5091.

## 2. Materials and methods

### 2.1. Cell culture

During the expansion and culture of mouse embryonic stem cell line, R1/E (ATCC, SCRC-1036™), the pluripotency of cells was maintained with feeder-dependent culture technique. Briefly, cells were cultured in Dulbecco’s Modified Eagle Medium (DMEM, Gibco, UK) supplemented with 15% embryonic stem-cell FBS (Gibco, UK), L-glutamine (Gibco, UK), 1× MEM nonessential amino acids (from 100× stock; Gibco, UK), 10 ng/mL recombinant mouse leukemia inhibitory factor (LIF, Abmgood, Canada) and 1% penicillin, streptomycin, and amphotericin B (PSA, Gibco, UK) on mitomycin C (8 μg/mL, Fisher Scientific, UK) treated mouse embryonic fibroblast (MEF) (ATCC, SCRC-1040) feeders. The cells were incubated in humidified incubator at 37 °C and 5% CO_2_ conditions.

R1/E cells were separated from MEF feeder cells to identify the effect of P5091 on the pluripotency of embryonic stem cells. mESCs and MEF cells were trypsinized, and the cell suspension was replated on a tissue culture plate. After 30 min of incubation at 37 °C, all MEFs were observed to be attached to the surface and the unattached, embryonic cells were resuspended in N2B27 medium (DMEM/F12 (Gibco, UK): Neurobasal Medium (Invitrogen, Gibco, UK) (1:1) supplemented with 0.5% N2 supplement (Gibco, UK), 1% B-27 supplement (Gibco, UK), 0.5% of 10% Bovine serum albumin (BSA) in phosphate buffer saline (PBS) (Sigma, USA), 1% L-glutamine (Sigma, USA) and 1% Antibiotic-Antimycotic (100X, Gibco, UK)) and plated on Matrigel (0.2μg/mL, BD Biosciences, Germany)–Gelatin (0.1%, Bioshop, Canada) (1:200) coated well plates. The pluripotency of mESCs was maintained by the application of two small-molecule inhibitors (2i) (3 μM CHIR99021 (CHIR, StemCell Technologies, Canada) and 1 μM PD0325901 (PD03, StemCell Technologies, Canada)) and 10 ng/mL LIF. Furthermore, M15 medium (Knock-Out DMEM (Gibco, UK), 15% fetal bovine serum (Gibco, UK) 1% Antibiotic-Antimycotic, 1% non-essential amino acids (100x, Gibco, UK), 0.1 mM β-mercaptoethanol (Sigma, Germany) and 10^3^ U/ml LIF (Abmgood, Canada)) was used to evaluate whether the 2i application alters the possible effect of P5091 on R1/E cells (Yang et al., 2017).

### 2.2. Preparation of small molecules

P5091 (Selleckchem, USA), CHIR and PD0325901 were dissolved in dimethyl sulfoxide (DMSO, Merck Millipore, Germany) to prepare 6 mM, 3 Mm, and 1 mM stock solution, respectively. CHX (Cycloheximide, AppliChem, Germany) was dissolved and diluted in M15 medium to a final working concentration of 50 μg/mL.

### 2.3. Determination of non-toxic P5091 dose

To determine the non-toxic P5091 dose on R1/E cells, 3-(4,5-dimethyl-thiazol-2-yl)-5-(3-carboxy-methoxyphenyl)-2-(4-sulfo-phenyl)-2H-tetrazolium (MTS) assay (CellTiter96 AqueousOne Solution; Promega UK Ltd., UK) was performed. mESCs were seeded on Matrigel-Gelatin coated 96-well plates (Corning, NY, USA) at density of 5×10^3^ cells/well. After overnight incubation, cells were treated with various P5091 concentrations (100 nM, 300 nM, 600 nM, 1μM, 3 μM, 6 μM) for 24 h. The viability of cells was measured by using PBS containing 4.5 g/L glucose and 10% MTS as described previously (Hayal et al., 2020). After 90 min of incubation in dark and humidified incubator, the viability was indicated *via* absorbance analysis at 490 nm.

### 2.4. Morphological analysis

mESCs were treated with the combinations of 2i, LIF and P5091 in N2B27 (or only P5091 treatment in M15 medium compared with M15 medium control) morphological changes of cells were observed under light microscope. Images were taken via ZEISS microscopy, AxioCam ICc 5 camera and ZEN 2 (blue edition) computer application. Additionally, the percentage of undifferentiated cells were calculated by using ImageJ software.

### 2.5. Alkaline phosphatase (ALP) staining

To analyze the change in pluripotency of mESCs, alkaline phosphatase staining (ThermoFisher scientific, Germany) was conducted according to manufacturer’s instructions. Briefly, mESCs were seeded on Matrigel-Gelatin coated 12-well plates (Corning, NY, USA) at density of 4 × 10^4^ cells/well. mESCs were treated with 600 nM P5091 in presence of 2i and/or LIF for 24h. To stain ALP in living cells, mESCs were washed once with DMEM/F12 (Gibco, UK). After washing step, stock stain (500 X) was diluted to the final concentration of 1 × with DMEM/F12. After 30 min of incubation at 37 °C, mESCs were washed three times with DMEM/F12 before imagining by using fluorescent microscope (ZEIS, AxioVert A1).

### 2.6. Quantitative real-time PCR analysis (qPCR)

Primer-BLAST software (National Center for Biotechnology, USA) was used to design USP7, SOX2, C-MYC, OCT4, KLF4, Brachyury, SNAIL, CDH1, GAPDH, WNT3a, Vimentin, Axin and β-Catenin primers (Table), which were synthesized by Sentegen. Total RNA was isolated using TRIZOL (Invitrogen, USA) according to manufacturer’s instructions and cDNA was synthesized by using iScript cDNA synthesis Kit (BioRad, USA). Quantitative real-time PCR was performed with CFX96 qPCR system (BioRad, USA) and SYBR green method was used to detect the alterations in transcription of target genes.

### 2.7. Western blot

Changes in pluripotency related Nanog, Oct4, c-Myc and Sox2 proteins were determined by western blot analysis. Additionally, successful decrease in de-ubiquitination activity of USP7 and alterations in Gsk3β and β-catenin proteins were also demonstrated by western blotting. Furthermore, the half-life of proteins was also determined after inhibition of translation by 50 μg/mL CHX treatment via immunoblotting (Lamberto et al., 2017; Ji et al., 2019). After each time point of CHX treatment, the cells were pelleted for protein isolation. Total protein was isolated by using RIPA lysis buffer system (Chem Cruz, Germany) according to manufacturer’s instructions. The proteins, each in 30 μg, were separated into their weight via SDS-Page electrophoresis and transferred to PVDF membrane (BioRad, USA). 5% Blotting Grade Blocker (BioRad, USA) was used to block the membrane for 1 h at room temperature. Nanog (1:1000, SantaCruz, USA), Oct4 (1:500, SantaCruz, USA), c-Myc (1:1000, Cell Signaling Technology, USA), Sox2 (1:500, SantaCruz, USA), Gsk3β (1:1000, Cell Signaling Technology, USA), β-catenin (1:1000, Cell Signaling Technology, USA) and ubiquitin (1:1000, Cell Signaling Technology, USA) specific primary antibodies were applied in blocking solution overnight at 4°C. After washing three times with Tris Buffered Saline-Tween 20 (TBST), membrane was incubated with proper HRP linked secondary antibody (anti-rabbit or antimouse, 1:10000, Cell signaling Technology, USA) for 2 h at room temperature and incubated with Amersham ECL Detection Reagents (Sigma, Germany) for 30 s. As an internal control, GAPDH specific antibody (1:10000, Cell Signaling Technology, USA) was used, and the images were taken by using the luminometer system (BioRad, USA).

### 2.8. Statistical analysis

All experiments were repeated at least three times, and statistical analysis was conducted by using one-way ANOVA and post hoc Tukey test. The statistical significance of P values (less than 0.05) was calculated by using GraphPad Prism 8.0 (GraphPad Software, La Jolla, CA, USA) software.

## 3. Results

### 3.1. P5091 treatment has no significant effect on cell viability

**Figure 1:**
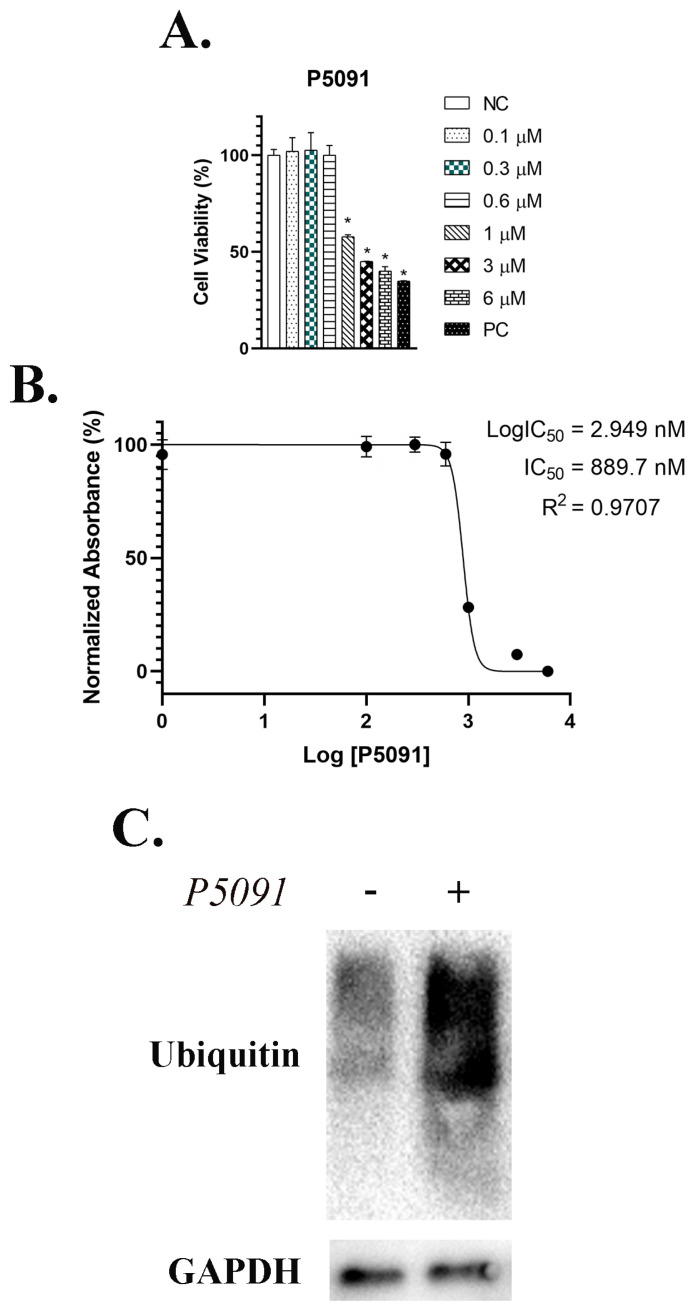
Non-toxic dose of P5091 inhibits USP7 enzyme activity in mESCs in vitro. A. Cell viability analysis of mESCs cells treated with various P5091 concentrations (0.1 μM, 0.3 μM, 0.6 μM, 1 μM, 3 μM, and 6 μM). No toxicity was observed for 0.6μM treatment. B. IC50 determination of P5091. 889.7 nM of P5091 treatment killed half of the mESCs in vitro. C. Total ubiquitination of mESCs with or without 0.6μM P5091 treatment. Amount of ubiquitin protein was found to be increased which indicates a successful inhibition of USP7 activity. Mean ± SD; n = 3 independent experiments; one-way ANOVA, *p < 0.05, compared with control group.

MTS assay was performed to identify the effect of P5091 on viability of mESCs. Various concentrations (100 nM, 300 nM, 600 nM, 1 μM, 3 μM, and 6 μM) of P5091 were applied to mESCs for 24 h. Results showed that application of P5091 at low concentrations (100 nM, 300 nM and 600 nM) did not affect the cell viability of mESCs (Figure 1a). However, 1 μM, 3 μM, and 6 μM P5091 treatments decreased the viability of mESCs by 42%, 54%, and 59%, respectively (Figure 1a). Additionally, half of mESCs were observed to be dead after 889.7nM P5091 application (Figure 1b). Furthermore, selected, non-toxic concentration (600 nM) of P5091 increased total ubiquitination levels significantly (≈ 12-fold) indicating the successful inhibition of USP7 enzyme activity by small molecule inhibitor, P5091, in vitro (Figure 1c).

### 3.2. USP7 inhibition effects the morphology and ALP activity of mESCs

**Figure 2:**
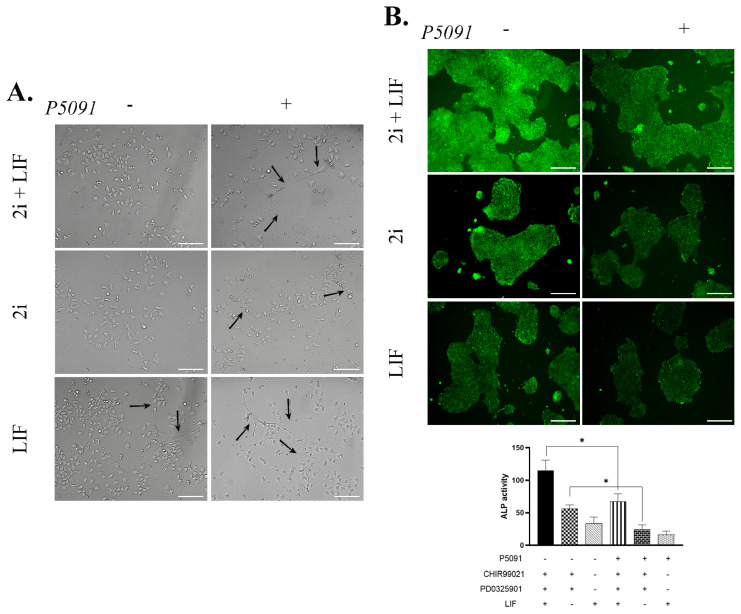
Inactive USP7 is responsible for decreased pluripotency of mESCs. A. USP7 inactivation changes the morphology of mESCs in vitro. Arrows indicate the morphological alterations of mESCs after 0.6 μM P5091 treatment for 24 h. B. ALP staining of all experimental groups indicates that USP7 inactivation decreases ALP activity, which is also correlated with pluripotency. Histogram shows the ALP activity in each group. Scale bar: 200 μm. 2i CHIR99021 (3 μM) and PD0325901 (1 μM) treatment, LIF Leukemia inhibitory factor, ALP Alkaline Phosphate. Mean ± SD; n = 3 independent experiments; one-way ANOVA, *p < 0.05.

To identify the impact of USP7 inhibition on mESCs morphology, cells were treated with 600 nM P5091 in the presence of 2i and/or LIF for 24 h on Matrigel-Gelatin coated plates and morphological alterations were determined by microscopic analysis. Results were determined that morphology of mESCs were affected by P5091 treatment. 2i and LIF application caused no significant change in cell morphologies of mESCs as expected. Yet, the addition of P5091 changed the morphology of mESCs to form spindle-like formation. Additionally, in every group, inactivation of USP7 resulted in impaired cellular morphology. Obtained results also showed that inhibition of ERK and GSK3β by 2i treatment and functional USP7 de-ubiquitination enzyme are required for pluripotency maintenance (Figure 2a). Furthermore, morphological analysis indicates that mouse embryonic stem cells might undergo differentiation after USP7 inhibition by P5091 treatment. Alkaline phosphatase (ALP) staining revealed that active ALP enzyme level decreased by 2-fold after P5091 treatment in 2i+LIF group. Similar effect was observed in 2i treated group (57% decrease in ALP activity). However, there was no noticeable change in the ALP activity in LIF treatment group (Figure 2b). Together with LIF application, the dual inhibition of GSK3 and MEK is also correlated with the pluripotency maintenance of embryonic stem cells. Thus, to evaluate whether the effect of P5091 on pluripotency maintenance is dual inhibition related, cells were grown in M15 medium without 2i application, and then they got analyzed for differentiated cells with the help of ALP staining. Obtained results indicate that the percentage of undifferentiated cells were dropped significantly (44%) after USP7 inhibition (Supplementary Figure 1).

### 3.3. P5091 treatment decreases the expression levels of pluripotency related genes

**Figure 3:**
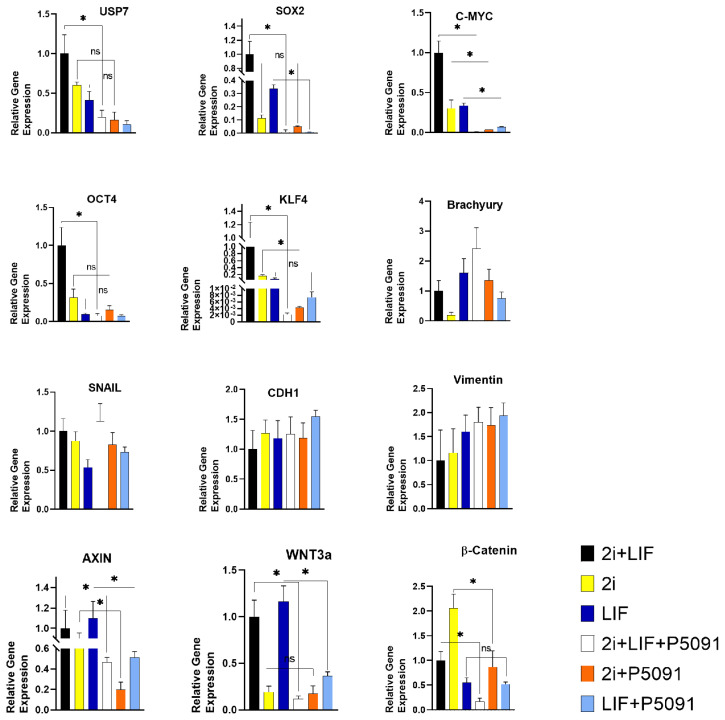
P5091 treatment decreased the gene expression levels of pluripotency related SOX2, C-MYC, OCT4, and KLF4 genes. However, no significant change was observed in mesoderm (Brachyury), ectoderm (Vimentin), and extra cellular matrix (SNAIL, CDH1) related gene expression levels. Furthermore, USP7 inactivation caused a decrease in expressions of AXIN, WNT3a, and β-Catenin genes. GAPDH mRNA was used as an external control. USP7 Ubiquitin Specific Protease 7, SOX2 SRY (sex determining region Y)-box 2, OCT4 Octamer-binding transcription factor 4, KLF4 Kruppel Like Factor 4, SNAIL1 Snail Family Transcriptional Repressor 1, CDH1 Cadherin 1, WNT3a Wnt Family Member 3A, GAPDH Glyceraldehyde-3-Phosphate Dehydrogenase, 2i CHIR99021 (3 μM) and PD0325901 (1 μM) treatment, LIF Leukemia inhibitory factor, mean ± SD; n = 3 independent experiments; one-way ANOVA, *p < 0.05.

To examine whether inhibition of USP7 has an effect on pluripotency, SOX2, OCT4, C-MYC, and KLF4 gene expression levels were determined in mESCs by gene expression analysis. Additionally, alterations in gene expression levels of mesoderm marker, Brachyury (T/Bra), ectoderm marker (Vimentin), extra cellular matrix (ECM) related CDH1 and SNAIL were also examined. Lastly, changes in gene expression levels of WNT3a and β-Catenin, which play a critical role in pluripotency homeostasis, were checked by qPCR analysis. All experimental groups were compared to relevant treatment groups including active or inactive USP7 protein to identify any possible change in pluripotency of mESCs. Gene expression profile of USP7 reduced by 10-fold after P5091 treatment in 2i + LIF group. A dramatic decrease was observed in gene expression levels of SOX2 (125-fold), C-MYC (142-fold), OCT4 (13-fold), KLF4 (320-fold), WNT3a (8.5-fold) and β-Catenin (6.25-fold) after inactivation of USP7 in 2i + LIF group. Similarly, in 2i treated group, P5091 application caused a significant decrease in C-MYC, KLF4, AXIN, and β-Catenin gene expression levels by 10-fold, 38-fold, 4.3-fold, and 2.5-fold, respectively. No significant change was observed in expression levels of USP7, SOX2, Brachyury, SNAIL, CHD1, and WNT3a genes. Also, USP7 inactivation reduced SOX2 (37.5-fold), C-MYC (5.5-fold), AXIN (2.12-fold), and WNT3a (3.2-fold) gene expression levels in LIF treatment group. Yet, there was no significant change in USP7, OCT4 and β-Catenin gene expression levels in LIF treatment group. Furthermore, noticeable change was not detected in gene expression levels of Brachyury, Vimentin, CDH1 and SNAIL in all experimental groups (Figure 3). The experimental setup was also replicated without 2i application, in M15 medium. Similar to 2i + LIF group, a significant decrease in SOX2 (17.8-fold), OCT4 (3.4-fold), KLF4 (5.4-fold), WNT3a (8.3-fold) and β-Catenin (1.8-fold) was observed (Supplementary Figure 1). Nevertheless, no significant change in C-MYC, Brachyury, SNAIL, and CDH1 was observed (Supplementary Figure 1).

### 3.4. Protein amounts of pluripotency related genes were altered due to USP7 inactivation

**Figure 4:**
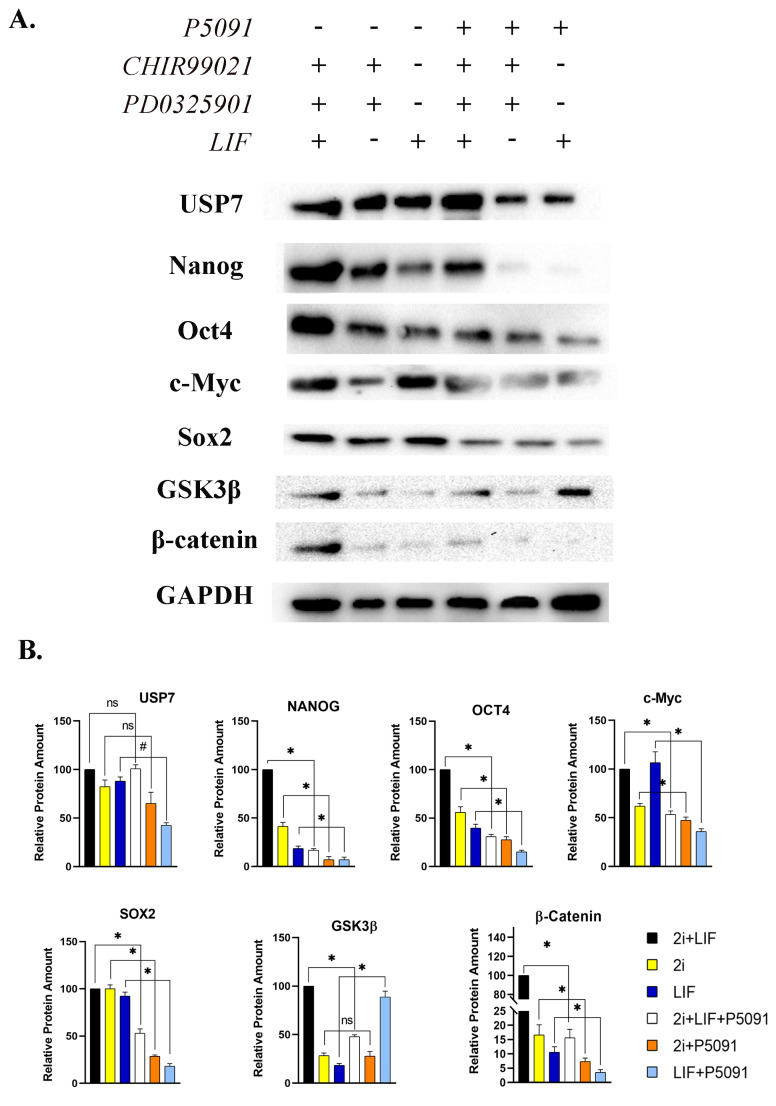
P5091 treatment decreased the protein levels of pluripotency related SOX2, C-MYC, OCT4 and NANOG genes. Inactivation of USP7 activity increased the protein amount of GSK3β and reduced the β-Catenin significantly. A. Immunoblot images taken by using imagining system. B. Band intensity calculations of various proteins relative to GAPDH. USP7 Ubiquitin Specific Protease 7, SOX2 SRY (sex determining region Y)-box 2, OCT4 Octamer-binding transcription factor 4, NANOG Nanog Homeobox, GSK3β Glycogen Synthase Kinase 3 Beta, GAPDH Glyceraldehyde-3-Phosphate Dehydrogenase, 2i CHIR99021 (3 μM) and PD0325901 (1 μM) treatment, LIF Leukemia inhibitory factor, ns not significant, mean ± SD; n = 3 independent experiments; one-way ANOVA, #p ≤ 0.001, *p < 0.05.

To further examine the effect of USP7 on pluripotency of mESCs, western blot analysis was conducted. Experimental compressions were performed on the same treatment groups with different USP7 activation status. P5091 treatment caused no change in USP7 protein amount significantly in ERK and GSK3β inhibited groups. Interestingly, USP7 protein levels were decreased by 50% after P5091 treatment in LIF treatment group (Figure 4). Nanog protein amount decreased in combinational 2i + LIF treatment group and individual 2i or LIF treatment groups by 82%, 33%, and 11%, respectively. Similarly, pluripotency related OCT4 protein level was found to be decreased in 2i (30%), LIF (24%) and combination of 2i and LIF (68%) groups after USP7 inactivation. Protein amount of c-Myc was also observed to be decreased significantly in 2i + LIF group by (47%), in 2i group by (12%) and in LIF treatment group by 70%. Correspondingly, amount of SOX2 protein also decreased after USP7 inactivation in all experimental groups by 50% (2i + LIF), 72% (2i) and 75% (LIF). Although GSK3β was inhibited by 2i treatment, USP7 inactivation in LIF treatment group resulted in a significant increase in GSK3β protein amount (72.3%). Furthermore, protein amount of β-Catenin reduced dramatically after P5091 treatment in 2i (9%), LIF (7%) and 2i + LIF (84%) groups (Figure 4). Overall, these findings suggest that USP7 enzyme activity not only maintains the pluripotency of mESCs but also protects β-Catenin from proteasomal degradation.

### 3.5. USP7 inactivation decreases half-life of GSK3β and β-Catenin proteins

**Figure 5:**
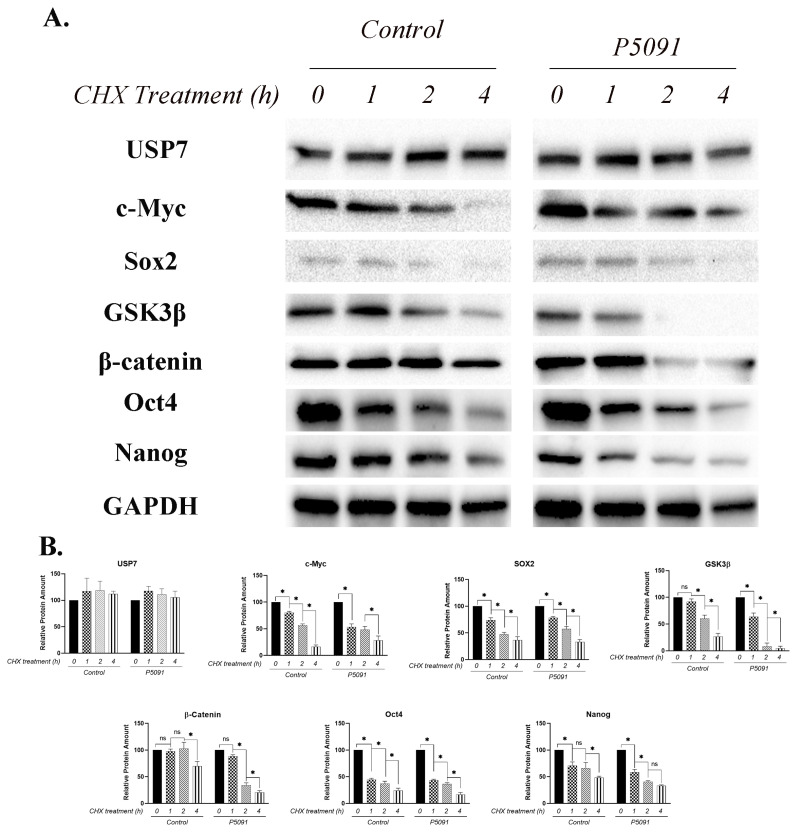
Blocked ribosomal translation via cycloheximide treatment shortens the half-life of GSK3β and β-Catenin proteins. Yet, no significant change was observed in the half-life of pluripotency related proteins after 4 h of treatment. The experiment was conducted in the presence of M15 medium. A. Immunoblot images taken by using imagining system. B. Band intensity calculations of various proteins relative to GAPDH. USP7 Ubiquitin Specific Protease 7, SOX2 SRY (sex determining region Y)-box 2, GSK3β Glycogen Synthase Kinase 3 Beta, GAPDH Glyceraldehyde-3-Phosphate Dehydrogenase, OCT4 Octamer-binding transcription factor 4, NANOG Nanog Homeobox, CHX cycloheximide, ns not significant, mean ± SD; n = 3 independent experiments; one-way ANOVA, *p < 0.05.

To evaluate the effect of USP7 inactivation on half-life of USP7, c-Myc, Sox2, Oct4, Nanog, GSK3β and β-Catenin proteins, the protein synthesis was blocked by CHX treatment for different time points (1 h, 2 h, and 4 h). GAPDH protein was used as an internal control. CHX treatment for 4 h did not affect the USP7 levels in both control and P5091 treatment groups. However, the protein amount of Sox2 decreased by approximately 15% after each hour in both control and P5091 groups. The amount of c-Myc protein decreased by 16%, 25%, and 35% after 1 h, 2 h, and 4 h of CHX treatments, respectively. However, no significant decrease was observed after 2 h of CHX treatment, one hour of CHX treatment cause a noteworthy decrease (35%) in c-Myc protein amount in P5091 treatment group. Unsurprisingly, the amount of c-Myc protein dropped significantly after 4 h of CHX treatment (15%) after P5091 treatment. A remarkable decrease in the protein amount of Oct4 was noted after 1h CHX treatment (approximately 64% in both control and P5091 treatment group). CHX treatment for 4 h causes a similar decrease in OCT4 in both experimental groups. Likewise, the protein amount of Nanog protein dropped coherently in both control and P5091 treatment groups. Additionally, the half-life of GSK3β and β-Catenin shortened after P5091 treatment. Although, 1 h of CHX treatment did not change GSK3β protein amount in control group, it caused a significant decrease (37%) in P5091 treated group. Correspondingly, 2 h and 4 h of CHX treatment decreased GSK3β protein amount even more dramatically after USP7 inactivation (after 2 h treatment, 30% and 55% decrease in control and P5091 treatment groups respectively; after 4 h treatment 32% and 40% decrease in control and P5091 treatment groups). Similarly, 1h and 2h of CHX treatment did not change β-Catenin amount in control group. Yet, USP7 inactivation cause a decrease in β-Catenin protein amount even after 1 h and 2 h of CHX treatment (by 12% and 50%, respectively). Also, 60% of β-Catenin remained functional in control group while 20% of β-Catenin remained functional in P5091 treatment group after 4 h of CHX treatment (Figure 5). These findings strongly suggest that USP7 inactivation shortens the half-lives of GSK3β and β-Catenin, disturbing the maintenance of pluripotency in mESCs.

## 4. Discussion

Post-translational regulation of proteins is usually controlled by ubiquitination which plays a significant role in almost every molecular pathway such as Wnt/β-catenin pathway (Gao et al., 2014) which is critical for pluripotency maintenance (Xu et al., 2016). The ubiquitination process is controlled by de-ubiquitination enzymes (DUBs), which can both block the ubiquitination sides of proteins and detach the ubiquitination tags from target proteins (Buckley, et al. 2012). As a DUB family member, USP7 is a perfect example for this kind of regulation (Nijman, et al. 2005). Therefore, in the current study, we aimed to identify the role of USP7 on pluripotency maintenance of mESCs. The impact of USP7 activation on pluripotency was evaluated after USP7 inactivation by small molecule inhibitor, P5091. Previous studies successfully identified P5091 as a specific molecular inhibitor for USP7 enzyme activity (Chauhan et al., 2012). The results demonstrate that high concentrations of P5091 show a toxic effect on mESCs. However, 600 nM P5091 treatment shows no significant effect on the viability of mESCs. Additionally, 600 nM P5091 treatment increases the total ubiquitination amount in mESCs indicating the successful inhibition of USP7 enzyme activity.

It is known in the literature that pluripotency maintenance can be observed by morphological analysis (Ye et al., 2017). Moreover, mESCs were assessed for ALP activity. The literature have shown that ALP activity is highly correlated with pluripotency of embryonic stem cells (Phay et al., 2021). Obtained results have demonstrated that allosteric inhibition of USP7 altered the morphology and ALP activity of mESCs in 2i or LIF treatment groups. It was previously stated that dual inhibitory application (GSK3 and MEK inhibitors) is required for morphological and pluripotency maintenance of mESCs (Petkov et al., 2014). However, inactivation of USP7 by 600 nM of P5091 treatment changed the cellular morphology and lessened the ALP activity of mESCs even in 2i treated groups suggesting the failure to maintain pluripotency without active USP7 enzyme. The reduction in the ALP activity of mouse embryonic stem cells also clarified that even in M15 medium, which supports pluripotency without GSK3 and MEK inhibitors (Yang et al., 2017), P5091 treatment is responsible for decreasing pluripotency.

Several studies have focused on transcription factors that are required for keeping embryonic stem cells at pluripotent state (Takahashi and Yamanaka 2006). The transcription factors, Nanog, Oct4, Sox2, c-Myc and Klf4, play an important role in both pluripotency maintenance and reprogramming of somatic cells into inducepluripotent stem cells (iPSCs) (Takahashi and Yamanaka 2006). Obtained results indicated that expression levels of pluripotency related SOX2, C-MYC, OCT4 and KLF4 genes decreased, but no significant change in mesoderm and ectoderm markers, T/BRA and Vimentin, respectively was observed after USP7 inactivation. This finding suggests that no significant change in ectodermal or mesodermal differentiation has been noted, although the inactivation of USP7 decreases the pluripotency. Gene expression analysis was also supported by Western Blot analysis. Decreased protein levels of pluripotency related Nanog, Oct4, c-Myc, and Sox2 transcription factors were observed. Previous studies have addressed the relationship between deubiquitinase activity and pluripotency maintenance (Suresh, et al. 2016). For instance, deubiquitination enzyme, USP21, can control pluripotency by stabilization of transcriptional factor, Nanog (Pei, 2017). Moreover, USP9X as a deubiquitinase was found to regulate early differentiation process of stem cells (Suresh et al., 2016). Also, USP22 regulates various pluripotency related transcription factors such as Sox2 and c-Myc (Suresh et al., 2016), which were observed to be actively expressed in human ESCs and iPSCs (Sridharan et al., 2009). USP7 was also correlated with the maintenance of stem cell properties. Huang et. al. conducted a study to identify USP7 and repressor element 1-silencing transcription factor (REST) relationship to increase neural stem/progenitor cell maintenance (Huang et al., 2011). In the current study, it was observed that USP7 inhibition also resulted in a significant decrease in WNT3a, AXIN and β-Catenin gene expression levels. It is known that USP7 stabilizes both Axin and β-catenin (Wang et al., 2019). This dual effect of USP7 makes it really unique enzyme to control Wnt/β-catenin pathway. Not surprisingly, protein amount of β-catenin was also found to be decreased in all P5091 treatment groups. M15 medium was also used to support these findings. It is known that Wnt/β-catenin signaling is affected by dual inhibition of GSK3 and MEK (Liao et al., 2018; Kwon et al., 2019). Thus, M15 medium was used to demonstrate the exact effect of USP7 inactivation on Wnt/β-catenin pathway. Obtained results were found to correlate positively with the dual inhibitory groups; the expression levels of pluripotency related genes as well as GSK3β and β-catenin expression were found to decrease dramatically due to P5091 treatment.

The effect of USP7 inhibition on pluripotency maintenance was further examined by half-life determination of pluripotency related proteins. The literature reveals that cycloheximide (CHX) treatment successfully blocks the protein synthesis in cells (Wittstock et al., 1993); thus, it can be used to examine the changes in half-life of proteins (Yang, et al. 2017). In the current study the protein synthesis of R1/E cells were inhibited by 50 μg/ml CHX treatment and alterations in pluripotency related protein half-lives were demonstrated by immunoblotting. It was shown in the literature that the half-life of USP7 protein was not affected by 50 μg/mL CHX treatment for four hours (Peng et al., 2019). Therefore, it can be suggested that observed alterations in half-life of proteins is due to USP7 inactivation. The result of this study reveals that USP7 inactivation significantly shortens the half-live of both GSK3β and β-catenin compared to control group. There was no significant change observed between control and P5091 treated groups in half-life of pluripotency related Sox2, c-Myc, Oct4, and Nanog proteins. These results show for the first time in the literature that USP7 plays a critical role in pluripotency maintenance by its ability to regulate β-Catenin stability.

Previously it was clarified that Wnt/β-catenin signaling is vital for pluripotency maintenance of mESCs (ten Berge et al., 2011). Unwanted differentiation of mESCs is blocked by the application of small molecule inhibitor of GSK3, CHIR99021, which works as an activator of Wnt/β-catenin pathway (Wu et al., 2013). Furthermore, LIF treatment triggers the JAK/STAT signaling pathway, which eventually inhibits the GSK3 (Ogawa et al., 2006). Dual inhibition of GSK3 and MEK is traditionally preferred in order to maintain pluripotency (Ying et al., 2008). Interestingly, there are several studies suggesting that inhibition of Wnt/β-catenin promotes pluripotency maintenance (Davidson et al., 2012). Thus, it is important to know the homeostatic role of Wnt/β-catenin pathway on pluripotent stem cells. It was shown in the literature that Wnt signaling pathway is required for proper differentiation, yet cellular adhesion related, CDH1 independent cytoplasmic activity of β-catenin is important for pluripotency maintenance (Sineva and Pospelov 2014). The results of the current study demonstrated no significant change in CDH1 and SNAIL gene expression levels after P5091 treatment. Therefore, a decrease in β-catenin gene expression level after USP7 inhibition might be correlated with the decreased pluripotency capacity of mESCs.

It was also demonstrated that USP7 plays a dual role in the regulation of Wnt/β-catenin pathway (Park et al., 2020). USP7 deubiquitinates Axin protein, which is a part of a complex that inhibits β-catenin protein. Thus, USP7 acts as an inhibitor of Wnt/β-catenin pathway (Ji et al., 2019). Contrarily, it is known in the literature that USP7 deubiquitinase activity rescues β-catenin protein from proteasomal degradation *via* its interaction with E3 ligase RNF220. Thus, USP7 also upregulates the β-catenin stability (Ma et al., 2014). These findings suggest that USP7 enzyme has a dual effect on Wnt/β-catenin pathway, which has a dual effect on pluripotency and stem cell differentiation. Therefore, USP7 enzyme activity, which is essential for post-translational protein regulation, is responsible for balancing pluripotency maintenance and differentiation decisions of mESCs in vitro.

## 5. Conclusion

Overall, USP7, as a deubiquitinase, might be very important for striking the balance between differentiation and pluripotency maintenance of mESCs. This study indicates that allosteric inhibition of USP7 decreases the levels of pluripotency related Sox2, Nanog, Oct4, Klf4 and c-Myc genes, and proteins through the posttranslational regulation of GSK3β and β-Catenin correlation in mESCs. Furthermore, USP7 inactivation significantly shortens the half-life of β-catenin protein. The real impact of USP7/β-catenin correlation on pluripotency maintenance of mESCs should be further investigated to clarify the exact molecular mechanism that underlies.

Supplementary Figure 1 USP7 inactivation decreases pluripotency of mESCs in M15 medium. A. USP7 inactivation changes the morphology of mESCs in vitro. B. P5091 treatment causes a significant decrease in ALP activity of mESCs in M15 medium. Histogram shows the decrease in ALP activity in each group. C. P5091 treatment decreased the gene expression levels of pluripotency related SOX2, OCT4 and KLF4 genes in M15 medium. However, no significant change was observed in mesoderm (Brachyury) and extra cellular matrix (SNAIL, CDH1) related gene expression levels. Furthermore, USP7 inactivation caused a decrease in expressions of WNT3a and β-Catenin genes. GAPDH mRNA was used as an external control. *USP7* Ubiquitin Specific Protease 7, *SOX2* SRY (sex determining region Y)-box 2, *OCT4* Octamer-binding transcription factor 4, *KLF4* Kruppel Like Factor 4, *SNAIL* Snail Family Transcriptional Repressor 1, *CDH1* Cadherin 1, *WNT3a* Wnt Family Member 3A, *GAPDH* Glyceraldehyde-3-Phosphate Dehydrogenase, mean ± SD; n = 3 independent experiments; one-way ANOVA, *p < 0.05, compared with the M15 medium control group.

## Figures and Tables

**Table:** Primer designs used in qPCR analysis.

Gene	Side	Sequence	Product Length
USP7	Forward	5′ CAGTCTGTAACCCGACTTCC 3′	184 bp
	Reverse	5′ CAGTCTGTAACCCGACTTCC 3′	
SOX2	Forward	5′ TTTGTCCGAGACCGAGAAGC 3′	146 bp
	Reverse	5′ CTCCGGGAAGCGTGTACTTA 3′	
C-MYC	Forward	5′ CGACTACGACTCCGTACAGC 3′	195 bp
	Reverse	5′ GTAGCGACCGCAACATAGGA 3′	
OCT4	Forward	5′ TGTTCAGCCAGACCACCATC 3′	100 bp
	Reverse	5′ GCTTCCTCCACCCACTTCTC 3′	
KLF4	Forward	5′ GAAGGGAGAAGACACTGCGT 3′	82 bp
	Reverse	5′ GGGGGAAGTCGCTTCATGTG 3′	
Brachyrury	Forward	5′ GTTCCCGGTGCTGAAGGTAA 3′	176 bp
	Reverse	5′ GCGAGTCTGGGTGGATGTAG 3′	
SNAIL	Forward	5′ CTCCAAACCCACTCGGATGT 3″	97 bp
	Reverse	5′ AGCCAGACTCTTGGTGCTTG 3′	
CDH1	Forward	5′ AACCCAAGCACGTATCAGGG 3′	94 bp
	Reverse	5′ GAGTGTTGGGGGCATCATCA 3′	
WNT3a	Forward	5′ CTCAGCTATGAACAAGCACAAC 3′	198 bp
	Reverse	5′ TGTTTCTCCACCACCATCTC 3′	
GAPDH	Forward	5′ AGGAGAGTGTTTCCTCGTCC 3′	187 bp
	Reverse	5′ TGCCGTGAGTGGAGTCATAC 3′	
Vimentin	Forward	5′ GCGAGAGAAATTGCAGGAGG 3′	112 bp
	Reverse	5′ CCGTTCAAGGTCAAGACGTG 3′	
AXIN	Forward	5′ CTCTGGGCTCAGGCTCC 3′	104 bp
	Reverse	5′ CACTCTCACCAGCCCTCTC 3′	
β-Catenin	Forward	5′ ATCATTCTGGCCAGTGGTGG 3′	192 bp
	Reverse	5′ AAGTCGCTGACTTGGGTCTG 3′	
