# Supplementary figures and images for: Ubiquitin specific protease 7 maintains pluripotency of mouse embryonic stem cells through stabilization of β-catenin
Source: Turk J Biol. 2021 Nov 17;46(1):82–94. doi: 10.3906/biy-2108-45 (PMC10393100; doi:10.3906/biy-2108-45)

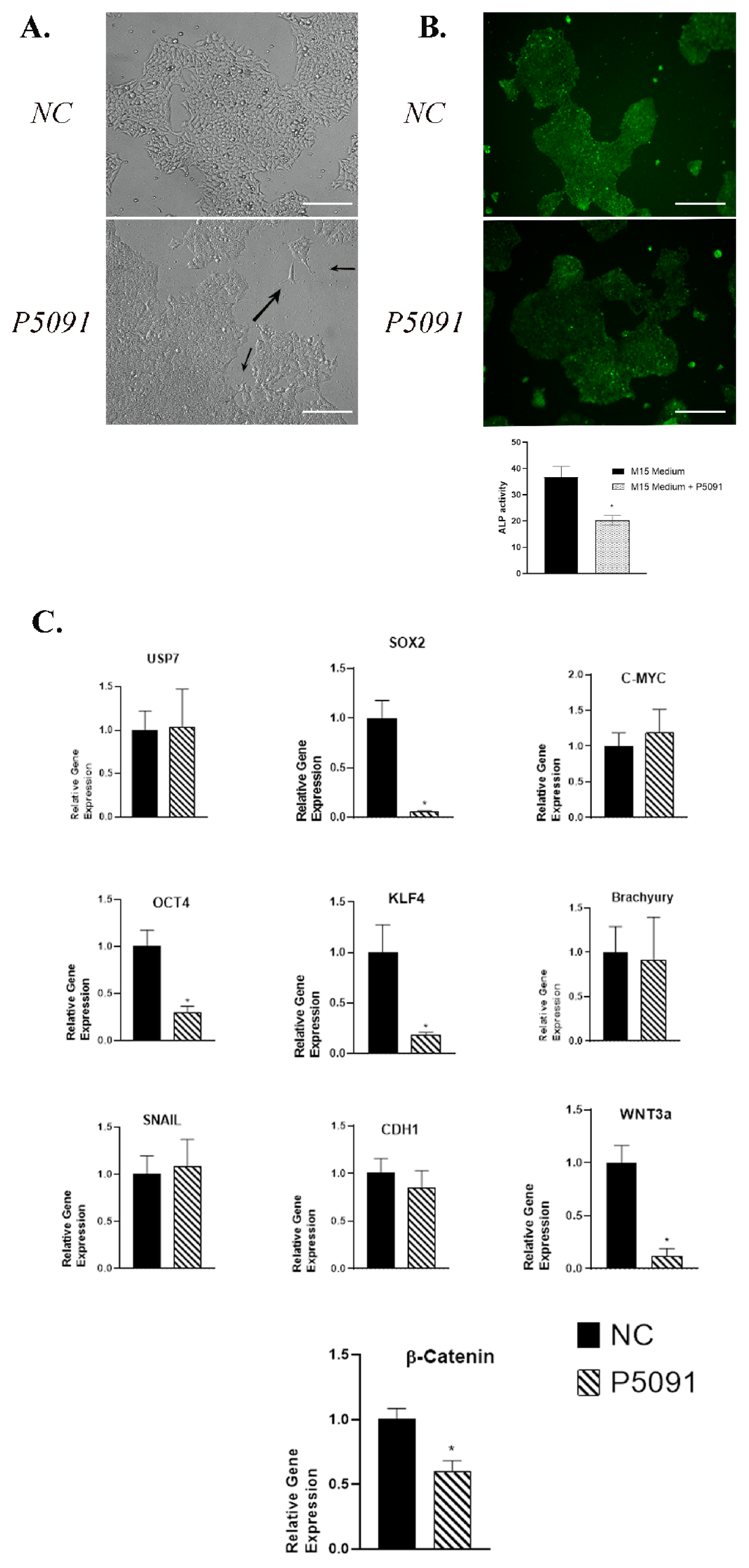

Supplement: Supplementary Figure 1 — USP7 inactivation decreases pluripotency of mESCs in M15 medium. A. USP7 inactivation changes the morphology of mESCs in vitro. B. P5091 treatment causes a significant decrease in ALP activity of mESCs in M15 medium. Histogram shows the decrease in ALP activity in each group. C. P5091 treatment decreased the gene expression levels of pluripotency related SOX2, OCT4 and KLF4 genes in M15 medium. However, no significant change was observed in mesoderm (Brachyury) and extra cellular matrix (SNAIL, CDH1) related gene expression levels. Furthermore, USP7 inactivation caused a decrease in expressions of WNT3a and β-Catenin genes. GAPDH mRNA was used as an external control. USP7 Ubiquitin Specific Protease 7, SOX2 SRY (sex determining region Y)-box 2, OCT4 Octamer-binding transcription factor 4, KLF4 Kruppel Like Factor 4, SNAIL Snail Family Transcriptional Repressor 1, CDH1 Cadherin 1, WNT3a Wnt Family Member 3A, GAPDH Glyceraldehyde-3-Phosphate Dehydrogenase, mean ± SD; n = 3 independent experiments; one-way ANOVA, *p < 0.05, compared with the M15 medium control group. [file turkjbiol-46-1-82s1.tif]
